# Multi-scale governance and data for sustainable development

**DOI:** 10.3389/fdata.2022.1025256

**Published:** 2022-12-01

**Authors:** David Pastor-Escuredo, Andrea Gardeazabal, Jawoo Koo, Asuka Imai, Philip Treleaven

**Affiliations:** ^1^Computer Science Department, University College London, London, United Kingdom; ^2^LifeD Lab, Madrid, Spain; ^3^International Maize and Wheat Improvement Center (CIMMYT), Texcoco, Mexico; ^4^International Food Policy Research Institute, Washington, DC, United States; ^5^United Nations High Commissioner for Refugees, Geneva, Switzerland

**Keywords:** networks, multi-scale systems, Artificial Intelligence, resilience, governance, Collective Intelligence, federated learning, policy

## Abstract

Future societal systems will be characterized by heterogeneous human behaviors and data-driven collective action. Complexity will arise as a consequence of the 5th Industrial Revolution and 2nd Data Revolution possible, thanks to a new generation of digital systems and the Metaverse. These technologies will enable new computational methods to tackle inequality while preserving individual rights and self-development. In this context, we do not only need data innovation and computational science, but also new forms of digital policy and governance. The emerging fragility or robustness of the system will depend on how complexity and governance are developed. Through data, humanity has been able to study a number of multi-scale systems from biological to migratory. Multi-scale governance is the new paradigm that feeds the Data Revolution in a world that would be highly digitalized. In the social dimension, we will encounter meta-populations sharing economy and human values. In the temporal dimension, we still need to make all real-time response, evaluation, and mitigation systems a standard integrated system into policy and governance to build up a resilient digital society. Top-down governance is not sufficient to manage all the complexities and exploit all the data available. Coordinating top-down agencies with bottom-up digital platforms will be the design principle. Digital platforms have to be built on top of data innovation and implement Artificial Intelligence (AI)-driven systems to connect, compute, collaborate, and curate data to implement data-driven policy for sustainable development based on Collective Intelligence.

## Introduction

The Data Revolution has achieved deep transformations in how United Nations (UN) as well as other multilateral agencies such as the World Bank work (Pulse, 2012, 2016; GSMA, U. G. P, 2017). However, this Revolution is still far from the population and is little immersive. Data and AI have been shown to act as catalyzers and enablers of Sustainable Development Goals (SDGs). They not only serve as the best approach to measure SDGs' targets and indicators but are also the best tools to face emergencies (Bullock et al., 2020; Luengo-Oroz et al., 2020). However, there are still scarce data to tackle all SDGs and their targets and indicators. We can argue that AI is mature enough to address the challenge of sustainable development (Discovery, UNGP; Vinuesa et al., 2020a). In recent years, we have witnessed the evolution of Deep Learning (LeCun et al., 2015), especially in convolutional neural networks (CNNs) (Li et al., 2016; Albawi et al., 2017; Zhang et al., 2019), Recurrent Neural Network (RNN) models like Gated Recurrent Units (GRU) (Dey and Salem, 2017; Shen et al., 2018; Shewalkar, 2019), and transformer-based approaches such as the Bidirectional Encoder Representations from Transformers (BERT) (Devlin et al., 2018; Acheampong et al., 2021; Yates et al., 2021). We have also witnessed lately the demand for Explainable-AI to present more transparent models that can be ethically and practically accepted in many applications (Hoffman et al., 2018; Holzinger, 2018; Gade et al., 2019).

The pandemic era has shown the need for new governance schemas. The lessons learnt from employing biological systems (Blanchard et al., 2009; Pastor-Escuredo et al., 2016; Pastor-Escuredo and del Alamo, 2020) and digital epidemiology (Salathe et al., 2012; Pastor-Escuredo, 2020b) can be applied to design new multi-scale governance. Tissue organization shows how coordination and signaling among cells leads to the robust process of organogenesis (Keller et al., 2003; Keller, 2012). Network science is also a powerful framework that is used to characterize spreading patterns (Boguná et al., 2003; Moreno and Vazquez, 2003; Balcan et al., 2010; Meloni et al., 2011; Salathe et al., 2012; Pastor-Escuredo, 2020b). By integrating dynamics systems, networks, and multi-scale systems, it is possible to design new governance frameworks.

In the era of the 5th Industrial Revolution and the Metaverse, we can expect different large-scale human processes that propagate across the tangled network created through digital connectivity, high-resolution media, world-wide economic and financial exchanges, and multi-scale mobility. Managing, designing, and leading governance constructs requires understanding and quantifying complexity as a previous step to exploit to create more resilient, robust, and thriving societies. Multi-scale governance will have impact on different sectors and industries as a game-changing catalyzer. The changes in industry and sectors along with new executive boards tools will allow scalable transformations for sustainability. Demographics and urban–rural development will also play a key role in governance changing livelihoods (Zufiria et al., 2018), risk (Taleb, 2019; Norman et al., 2020), inequalities, and trade.

The data ecosystem that is to be built is different from the data ecosystem that was used during the first era of the Data Revolution. Use case-driven data sharing agreements are focused on innovation and research, but they have not scaled up to change society. Digital platforms and data gateways have to be the basis for multi-scale systems based on complexity. This perspective presents how data-driven governance can help build a resilient and sustainable world. Integrating public, non-governmental, and private governance is necessary to leverage all the Big Data and Artificial Intelligence (AI) that the society can generate and is still unlocked. Federated systems need to be built as the basis of multi-scale governance to share data, reports and maintain leverage from all stakeholders. We envision a public Metaverse fed by “Phygital” spaces that are immersive to produce in- situ Big Data that are necessary to measure and achieve the SDGs. We present the elements of multi-scale governance for a multi-scale and multi-level complex system-based society (Omodei et al., 2022).

## Future of digital governance

Transformative processes require drivers and catalyzers to generate phase transitions and enable structural changes. These are the constitutive elements of multi-scale governance. Most of the current organizations and governance structures are stiff. Dynamic organizations must replace current organizations to be more sensitive and responsive to external phenomena, either positive or negative, to increase synergies and make good decisions.

Early warning mechanisms must be at the core of governance in private, public, and social sectors. Although Artificial Intelligence (AI) and Data have been used as early warning systems (Kryvasheyeu et al., 2016; Wilson et al., 2016; Pastor-Escuredo et al., 2020) in humanitarian contexts, there is still a big gap in implementing such systems in corporate governance and public management. Furthermore, besides issuing alerts, governance requires simulations to understand the effects and impact of actions to be taken or to design response in real-time but with clear response priorities. Dynamic organizations are more resilient and help build a more resilient and robust society and socioeconomic tissue.

Coupled with alert systems, multi-scale governance requires to set long-term objectives that are properly articulated with real-time response and decision-making. Sustainability Development Goals (SDGs) are a proper framework to establish mid- and long-term objectives that are holistic and allow sectors to interact and find synergies to drive toward a more sustainable society. For these reasons, dynamic processes have to also be led by governments, multi-lateral actors, and international institutions in collaboration with private sector corporations.

Regarding the spatial and human scope of governance, holistic frameworks powered by complex science used within multi-stakeholder ecosystems are the way forward to create an evidence-based governance that is effective for causing a high socioeconomic impact. Artificial Intelligence (AI) is used to get insights based on data, but normally in a closed environment and with limited socioeconomic scope. Private sector governance will not help change this trend, as privacy and competitive interest hamper sharing data at scale.

Federated learning (Yang et al., 2019a) is a potential solution to manage privacy and competitive interests through decentralized access to data and algorithmic agents. However, centralization of indicators is also required to generate transversal insights that are necessary for policymakers, public leaders, new innovators and entrepreneurs, and different types of social and humanitarian agencies. The proper solution to implementing such a centralization process will rely on different levels of utility-privacy where the data can be queried depending on its potential positive impact, mitigating privacy risk, and augmenting social benefits (Pastor-Escuredo et al., 2020).

Data sharing and federated learning are key, as future governance requires a transition to generate wide insights that open perspectives and encourage collaboration and transformative projects. Machine Learning (ML), data science, and visual analytics should evolve to create these knowledge-based ecosystems that are not only sound scientifically but also have a high social value. Depth and wideness are two elements that need to be to be promoted in decision-making processes through learning and training of decision makers and leaders.

Evidence-based policymaking and AI-driven systems also require ethical frameworks to be acceptable and useful. An example of such an ethical framework is the use of digital apps for contact tracing that have given rise to social and ethical concerns that depend on the technological design, their deployment, their governance, and their application (Altmann et al., 2020; Berman et al., 2020; Vinuesa et al., 2020b). Human-centered design is required, but it should also be seen from a broader perspective of an acceptable digitalization both in terms of technology and technology governance (Pastor-Escuredo, 2020c). Typical technical values, such as transparency, efficiency, and trustworthiness, are necessary, but not sufficient to be the ground for evidence-based policymaking in the digital era. Ethical frameworks will depend, of course, on cultural particularities, but it is important to have global frameworks for governance for a more coordinated society that can achieve sustainability development goals. Beyond evidence-based decision-making, the digital technology can help in building more distributed, responsive, flexible, and accountable systems for policymaking.

## Structure of data-driven governance

We can identify different levels within the society that is seen as a complex system. Beneficiaries are the first level and the ones who benefit more from direct and indirect AI innovations, as SDGs comprise specific targets for beneficiaries. There is not yet any standard mechanism for beneficiaries to produce exhaustive data beyond mobile phone Call Detail Records (CDRs), so we should expect the introduction of such services in the 5th Industrial Revolution. Data from beneficiaries should still mostly be managed by the UN and partners, but we have not witnessed an exponential growth in beneficiaries' data, either data or a digital economy that empowers beneficiaries. Still, beneficiaries' data are the most valuable asset for non-governmental and governmental agencies to deliver sustainable development and resilience systems.

Population is the second level and the one that leverages more shares of the global AI innovation and creates incentives for all other levels. Population is the biggest producer of data, however the data are not fully geolocated but disaggregated to tackle any humanitarian work or SDG-related projects. We need to go in depth into the design of an ontological system that allows the collection of disaggregated data for SDGs and helps to connect beneficiaries and population (i.e., studies on social and residential segregation). Meta-population is the third level and the one that can be more actionable, as they have more specific resources that are allocated for SDGs' targets (i.e., types of migrants). Meta-population data are useful to track the impact of humanitarian work and SDG projects as the traceability of specific population is the best known way to generate the ground-truth for humanitarian evaluation and to design humanitarian systems.

The fourth level is the private sector that is the one sector that generates more AI innovations and collects more amount of data. However, they have a little role to play in SDGs, as there are not sufficient incentives and mechanisms for participation in SDGs. Private sector data have not been fully leveraged, and this includes specially unstructured data that contain all works on sustainability from the private sector. In a growing development work, private sector will still the principal partner to leverage data for beneficiaries, population, and meta-populations.

The fifth level would be comprised by digital platforms, mainly conformed by Academia, but more connected with the private sector and professionalized for multi-scale governance and SDGs. Digital platforms would not only help with research for innovation, but also with advocacy, data infrastructure, and management of intelligence. Finally, the last level would still be composed by non-governmental bodies such as multi-lateral banks, UN, and large research and action on-the-ground centers for development. We have witnessed an important internal change in government's and non-government's infrastructure to collect data that have to go further on to develop more scalable data systems [i.e., collaborative United Nations Children's Fund (UNICEF's) Magic Box].

The AI can help manage this ecosystem through agent-based modeling and simulation tools based on Reinforcement Learning (RL) and Generative Adversarial Networks (GANs). Also, early warning systems can be leveraged to track progress in SDGs and activate the networks. Furthermore, a multi-level structure of sustainability allows the generation of data to measure impacts and secondary impacts of crises, emergencies, and epidemics that are not available now. Quantifying internal system structure and dynamics interlinked with external phenomena is the key for boards and decision makers.

In this regard, multilayered networks (De Domenico et al., 2015; Aleta and Moreno, 2019) are a proper tool to model interactions between intra- and interorganizations. Data are the missing element to populate these models. Having structured data of organizations' interactions would allow computation to better model how sectors can cooperate, how organizations can implement structural changes, how economy can be better stimulated, etc. Federated learning will allow us to progress toward interconnected socioeconomic networks for data-driven governance.

Data and AI innovation has shown to be the best enabler and catalyzer of sustainable development. This proves the success of the Data Revolution. A 2nd Data Revolution is needed across all SDGs to keep progressing. A 2nd Data Revolution should be based on complexity and multi-scale structures to scale-up the impact and speed of Data and AI-driven development.

The structure of a multi-scale system comprising several societal levels requires new enablers beyond mobile phone data, which were seen to be the major enablers in the last decade. Industry 5.0 is now becoming a new reality, demanding more immersive experiences and more ubiquity in data generation and use. Through Industry 5.0, we will be able to reach those many spaces where no data are available and so AI could not be deployed. As part of the immersive trend, Phygital spaces will help in acquiring data beyond the weary Internet of Things (IoT) ecosystem. Phygital shops have been one of the first examples of new digital environments that can be extended to cities, parks, fields, etc. Phygital generates data at the individual level within an immersive ecosystem, so it is suitable to understand the behavioral patterns that so far can only be inferred from mobile phone data use and credit card transactions. Beyond, the Metaverse is also becoming a reality that the world demands. Metaverse will also be a more immersive experience that the current Internet cannot be exploited for SDGs, apart from scarce health-related studies based on Google searches. The Metaverse will also take social media studies further, as we can expect more connected profiles in social media where data can be connected across different platforms. Social media made an important contribution to the Data Revolution that has decayed because of the lack of global positioning system (GPS)-referenced data and the appearance of infodemics that hamper serious studies based on platforms such as Facebook and Twitter. Metaverse is a great opportunity to revitalize social media data connecting platforms and gives rise to new platforms and social media apps where data of more specific SDG targets can be gathered. Specific SDG-related apps can appear in the context of Metaverse and provided incentives for digital, SDG, and data economies. Phygital spaces are also a gateway to the Metaverse, so the connections to it are not just constrained to a digital realm, but also a physical realm that is immersive.

More enablers are necessary in terms of technological infrastructure including ubiquitous 5G to generate more streams of Detail Records with access to the Internet [extended detection and responses (XDRs)]. Not only that development, but new infrastructure regarding data storage and algorithms tracing is necessary. Meta-databases of algorithms, models, and indicators are necessary to keep track of the data used (including type of data, resolution, provider, version, aggregation, anonymization, etc.) and the algorithms and workflows used (version, developer, libraries, etc.). Meta-databases are necessary to generate the accountability and reproducibility within the Data Revolution for sustainable development. We can find examples of meta-databases in computational music retrieval or computational biology including open data repositories and models' libraries or semantic web-based search engines.

Finally, more development-specific meta-structures are also required including indicator dashboards. To date, very few studies have addressed meta-models based on indicators because of the lack of dashboards and databases. High temporal and geographical resolution indicator histories are necessary to evolve the models for sustainable development and drive digital policymaking, which is restricted to internal workflows within the UN agencies. Local and global indicator dashboards should be part of the digital ecosystem including new elements such as the Metaverse.

Other meta-structure required is a system for impact–investment assessments. We have sufficient AI tools including transformers (Devlin et al., 2018) to infer temporal relationships between financial tools and investment and impact. Impact can now be measured through Natural Language Processing (NLP) and Machine Learning (ML) techniques to extract ground-truth from reports, roadmaps, and agendas and also indicator dashboards and social media narratives. Relationships between investment and tracked impact can be deciphered in a similar fashion to semantics interpretation and tagging by time series analysis and RNNs including Long Short-Term Memory (LSTM), GRU, and transformers [i.e., BERT, RoBERTa (Robustly Optimized BERT Pre-training Approach)]. Investment modeling can be tackled through Reinforcement Learning (RL) (Sutton and Barto, 1999; Wiering and Van Otterlo, 2012; Li, 2017; Henderson et al., 2018) and Generative Adversarial Networks (GANs) (Liu and Tuzel, 2016; Metz et al., 2016; Mao et al., 2017; Wang et al., 2017; Creswell et al., 2018; Gui et al., 2021), which have provided the sufficient amount of indicators with a high temporal resolution and a narrow geographical resolution. Impact—investment tools must work at the level of targets, so it integrates several SDG indicators, and imply several interconnections between SDGs.

In this context, we can expect the appearance of new data sources to come from a new Industry that is not only connected but is immersive and multi-modal, for instance, new data sources of responsible consumption that cannot be leveraged, as clothing companies do not have the necessary help to leverage their data.

To scale-up and speed-up, the system must comprise catalyzers. Catalyzers include traditional strategies, such as global Challenges, which have however become very scarce since the mid-2010s. These catalyzers also incorporate data for social good programs in all companies. However, this role has not been assigned any regular position within companies. Digital platforms can help in defining strategies for social good within companies with relevant data that cannot create their own data for a social good program. Thus, we can expect more Challenges with new data and after integrating different data sources (i.e., UN Data for Climate Action Challenge).

Another type of catalyzer includes new data gateways, which have provided standards and libraries that help in creating gateways systematically. Digital platforms can assume a great part of the social good responsibility, as companies do not have the necessary resources to implement data gateways for social good, as it has been showed that non-governmental agencies do not pay for data uses. Furthermore, financial resources for this purpose apply only in the case of large Hackathons that companies cannot organize alone. Infrastructure is necessary, apart from gateways. Federated learning and Blockchain are useful tools to implement digital and data economies. Traceability of data transactions is a powerful FinTech Incentive for companies to provide assessments of impact of their contribution and digitalize the platforms and data projects for social good.

Rights are also an important catalyzer for Data Revolution. Recently, we have evinced keen interest in enumerating digital principles (Jobin et al., 2019; Luengo-Oroz, 2019). A comprehensive framework of ethics in the digital era (Pastor-Escuredo and Vinuesa, 2020) can help create incentives and forces to drive digitalization and the AI work to make sure that AI plays an enabler and catalyzer role and it does not have negative effects (Vinuesa et al., 2020a) that hinder digital sustainable development.

SDGs' targets conform to a complex interconnected system where targets can overlap their action over societal elements. In this context, it is easier to exploit complexity and data science to create tools that enable synergic work and integrative policies. SDGs' targets can aim at beneficiaries, the development system, macro-economics, and finance. Thus, SDGs' targets are at a suitable abstraction level to work with AI and algorithms rather than at the SDG indicator level where the analysis ends up being a data aggregation process.

Beneficiaries are active and passive receptors of data and AI innovation for SDGs. Luckily, overlapping innovation on beneficiaries is always constructive but not always synergic. Cross-innovation of AI for different SDGs and their targets can help increase the speed of development in many places of the world. The AI-driven systems for development must also be synergic to avoid inhibitor roles of the AI, considering the complexity of targets regarding social and gender equality, land and climate complex processes, and green economy. It is also necessary to account for the reduced set of financial tools for SDGs of a world that has to recover from crisis and pandemics. It is hard to justify investment in SDGs that cannot be oriented in a holistic way and for ensuring that there are no negative effects such as digital gaps. Thus, impact–investment tools are necessary for advocacy and investment tracing and stimulation. It is, therefore, necessary to account for the investment of SDGs in the global macro-economy.

## Networked intelligence and incubators

Superminds (Malone, 2018) are a novel paradigm to solve complex problems combining both humans and machines. We are moving toward other forms of intelligence by leveraging Artificial Intelligence (AI) in unexplored ways. Such systems require a novel design based on AI, networks, and meta-structures of governance (Pastor-Escuredo and Treleaven, 2021). Networked Intelligence is a type of intelligence that emerges from large-scale networks and provided Collective Intelligence mechanisms to manage ideation, connection, evaluation, curation, synthesis, and governance. Networked Intelligence platforms are based on decentralized and federated technology to keep traceability of communication, transactions, idea flows, and narratives (Pastor-Escuredo, 2020a). The SDGs can be addressed by using Networked Intelligence to generate the necessary ground-truth and rich dynamic data to deploy AI and ML for SDGs' targets and indicators (Vinuesa et al., 2020a).

Incubators integrate complex governance and collaboration with AI tools and digital infrastructure based on groups of specialized people that generate emerging intelligence within the scope of action. Incubators can be a type of Supermind when the AI is used to self-govern the incubator and extract knowledge in real-time. On the other hand, Superbrains are based on networks of people with a regional scope where knowledge flows across the networks and AI is used to understand the dynamics, knowledge, and innovation of the ecosystem.

### Networks and superbrains

When the Networked Intelligence has a topology (network structure and shape) and also a topography (a geographical distribution), we call it a Superbrain. This feature can exploit the topology and dynamics of complex networks and network of networks to drive innovation and generate actionable knowledge for policy. A Superbrain captures the geographical variability that is reflected on social media and the behavior of ecosystems and digital ecosystems in different parts of the world.

Understanding the dynamics of this network is somewhat similar to understanding the dynamics of a brain that is based on connectivity. Network analysis is capable of deciphering the structure, topology, and dynamics of geographical networks. Topology and structure can be designed and analyzed using centrality metrics (betweenness, closeness, eigenvalue, and degree centrality). Through centrality metrics, we can monitor and govern Networked Intelligence and adjust it for different purposes. On the other hand, dynamics of the network can be analyzed through percolation and diffusion metrics.

Content and knowledge through the network can be analyzed using Natural Language Processing (NLP) and ML which implies novel uses of these techniques that, in combination, are currently limited to text mining. Semantics analysis on top of the network can help in creating a more sophisticated topic tracking analysis in an interconnected way (Pastor-Escuredo, 2020a). Furthermore, it can help track SDGs by looking at the truth of SDGs' indicators by world-mining not only through social media and indicator dashboards but also through unstructured data from private corporations within the network (Environment-Social-Governance – ESG- and Sustainability reporting). A step further is to perform narratives' analysis (Pastor-Escuredo, 2020a) where the dynamics of semantics and the dynamics of the network are jointly analyzed. This analysis is useful to understand change and transformations driven by SDGs and measure resilience.

Finally, the networked intelligence has to be monitored over time with tracking metrics to measure the impact of policies, summits, and global initiatives including funding. This is a necessary step to evaluate impact–investment for sustainability policies and SDGs' implementation and acceleration.

In terms of structure, a Superbrain should expand an aggregated network on the scale of 10,000 members including at least four geographical regions. The network must contain high-closeness to have regions of through-generation beyond clusters or small groups “design-thinking” and have consistency and diversity. The network also requires high-betweenness to have flows of information and thought for knowledge exchange, coherence, and consolidation (Pastor-Escuredo and Frias-Martinez, 2020). The role of leaders should be investigated by parametrizing the eigenvalue centrality of the network and the impact assessments (Pastor-Escuredo, 2020a).

In this context, new AI can be developed to assess diversity and richness, measure socioeconomic and gender equality, summarize activity in the network, and measure the impact of public and non-governmental actions on the socioeconomic tissue that is necessary to measure the SDGs.

### Digital incubator

A digital incubator is basically conformed by people and students organized as a governance tool beyond the concept of crowdsourcing and massive surveys. A digital incubator integrates humans and digital tech such as Blockchain and AI to change policy- and decision-making processes. Incubators can complement an expert-driven policy. An incubator has to collect data massively and make people within the incubator part of the data generation processes. Data can be collected through surveys and validation schemas, through crowdsourcing, through phygital spaces with enough sensors and communication channels to create immersive experiences, and potentially through Metaverse.

Machine Learning (ML) has to digest data and knowledge generated within the incubator and create representation spaces where the analyzed the data have to go back to the people within the incubator through real-time tools and dashboards. AI has to be used for managing decision-making within the incubator as an organized crowd. Furthermore, it has to evaluate and measure the performance of the incubator over time.

In terms of the structure, a digital Incubator can be implemented as a networked-Supermind (Giacomelli, 2020). The incubator should be composed by a network of people of around 1000 members that can grow in later stages. To define the topology of the networked-Supermind, we propose parametric configuration based on centrality values (Pastor-Escuredo and Frias-Martinez, 2020). The networked-Supermind requires a low level of closeness centrality per node to avoid self-feedback on information and creativity. However, it requires a high level of betweenness–centrality for information to flow and have good updates from information (data) feeders in the Supermind. Eigenvalue–centrality can be used for different setups and configurations of the Supermind. The configuration and topology should be visualized and monitored with network visualization. The networked-Supermind would interact with external networks and AI should be used to automate the functioning of the Supermind.

#### Network illumination

The selection of nodes can be made through network analysis based on topology and centrality of the networked-Supermind and its nodes. Concretely, node selection for a specific policy issue could be done by measuring the eigenvalue centrality of the nodes (Pastor-Escuredo and Frias-Martinez, 2020).

#### Energize

Incentives and goals in the digital era should be extracted in an automated way from external networks including the analysis of narratives from social media and external unstructured data (policy reports of think-tanks and other institutions). This comprises the analysis of influencing external networks nodes and also semantics analyzed with NLP.

#### Information feeders

Information feeders can be automated by data scrapping up to a large extent. It has to follow a federated-ML and federated-data structure based on Blockchain along with meta-databases to keep track of transactions. Public–private data can be resolved through a federated architecture where algorithms extract information from multiple thought and data providers. The federated architecture is built using a meta-layer of data processing comprising NLP, analytics, networks, time series, electronic surveys, etc.

#### Wire

The collaboration environment is also a digital meta-structure enhanced with AI with the following features: idea generation support (i.e., ideas mapping and priorities) and tracking of ideas based on indicators and impact assessments.

## Real-time and federated data and AI

From federated technology to collective action and decision-making, we can exploit AI and ML technology that is currently available and little used for decision-making processes. Federated data infrastructure (Bonawitz et al., 2019; Yang et al., 2019a,b; Li et al., 2020; Treleaven et al., 2022) across digital platforms including data gateways is the most scalable and trustful way to unlock public–private data for SDGs beyond data sharing projects (Pulse, 2015). Private governance still needs mechanisms and incentives to deliver structured Big Data and unstructured data (reports, agendas, etc.) for SDGs. A comprehensive data ecosystem requires multiple companies from all sectors to make data available in such a way that is acceptable for boards and stakeholders (Pastor-Escuredo and Treleaven, 2021).

New systems based on federated platforms built on top of Blockchain could definitely help for better decision-making processes that ensure ethics, responsibility, sustainability, and resilience in such a way that they can be measured and evaluated over time. Corporate governance has not jumped into SDGs globally because there are few incentives and no standard platforms to do so. We could not even witness a global platform for CDR data in the past decade, as it was the sector that drove the Data Revolution. Federated platforms imply several benefits including privacy, standard algorithmic anonymization and aggregation, reduced costs in data storage and data migration, less legal barriers for corporations, responsible jobs for teams working on Big Data technologies and Data Lakes, less barriers for executive boards to leverage internal data with competitive interests, etc.

The demands of a distributed system for a networked-data ecosystem are relevant when considering connections at the global level and the need to update the data and indicators. Indicators and data aggregates must be re-processed every 2 weeks if not in real-time in case decision makers trigger the process or an emergency appears. During emergencies, the agencies and development system may require a weekly update. This implies massive data analysis on a daily basis with slots of 2 weeks to update models and processing pipelines. The amount of data can only be reduced with data aggregates if spatiotemporal resolution is suitable, which implies the majority of the analysis that is performed nowadays. However, socioeconomics and human mobility imply high-resolution data and processing pipelines that may take up to 2–3 days to process.

The case of Early Warning is more complex in terms of management, as false positives and negatives are not acceptable in general. Complementary to data analysis, there exist tools such as Rapid-Pro from UNICEF that can trigger surveys and rapid text messages to confirm crisis situations or conflict. However, there is still a lack of digital protocols. High mobility groups and specialized teams from the United Nations Office for the Coordination of Humanitarian Affairs (UNOCHA) can also be triggered by a data-driven and AI-driven protocol in the nearby future, always in collaboration with other agencies. Digital protocols must include the integration of surveys and Small Data through baseline protocols to align all data in a timeline, so that Big Data and Small Data can be integrated and contrasted.

Computational complexity can only be solved by Global Data Science Networks where data scientists can actively share models and development of their frameworks. Meta-data science may not be robust enough for sustainable development, although it is necessary to contrast epistemology and ethics issues including machine behavior. Innovation is also relevant. Human-in-the-loop has worked on a report basis (both slide presentation and pythonic web), not as algorithmic workflows. This implies an expected 10-day latency in the loop. This can be aligned with a 2-week basis of model development. More sophisticated forms of Collective Intelligence within organizations are necessary for sense-making and decision-making based on the massive amount of data-based knowledge that emerges from computational pipelines.

Considering the tangled multi-level network dynamics and properties, real-time analysis and governance is key. A very basic step is to have shared spaces for knowledge gathering such as Teams where the information can be updated in nearly real-time. Common spaces are necessary along with real-time decision-making tools (i.e., traffic light priority maps). Vertical internal data flows based on data science are necessary, including reports and other more automated solutions such as Corporate Dashboards and internal signaling based on mobile phones and chats. We can consider that data science teams need to play facilitator roles to transform data into corporate decisions and make sure that agencies work real-time and that information is processed across layers externally and internally. Digital platforms can also play that role outside agencies and facilitating public–private collaboration and data gateways.

Of course, centralized platforms such as UNICEF's Magic Box are still necessary for exhaustive data science and AI research, policy reports, fast actionable analysis, response to crisis, and complex data integration. Federated algorithms are complement to centralized data science to explore new data sources in a scalable way. Centralized data and computation hubs for analysis of the dynamics of multi-level networks are required including the metrics of Early Warning and network dynamics (propagation and percolation), as it has been shown during epidemics. Centralized systems are necessary to keep consistency and coherence of the global data ecosystem and in enforcing timing constraints.

Transfer learning has been conceived of as a way to solve the problem of scarce data repositories across the globe for algorithm training and as technological innovation. Transfer learning should be able to deal with a set of characteristics: behavioral patterns, infrastructure types, and land colors. Thus, CCN-based algorithms are difficult to use in a transfer learning context. We need more science on building data repositories beyond challenges to be able to assess better transfer learning and smoothly integrate training data and pre-training. Epistemology work is lacking in the field of transfer learning and the creation of data repositories. National-level repositories are necessary for satellite data [Hang Seng Index (HSI) data], mobile phone data aggregates, or credit-card aggregates to deploy transfer learning for sustainable development. Feature-level learning with heterogeneous data has mainly been tested with CNNs and has the potential to obtain poverty indicators and population indicators if contextual info to buildings can be integrated. Collaborative work is also necessary within digital platforms to scale-up the number of analysis and tackle the tracking of SDG's target and indicators over time.

## Conclusion

Societal challenges demand digital technology to drive policy beyond Think-Tanks. For this purpose, it is necessary to design new systems and digital platforms encompassing digital tech and people that are also actionable and accountable like Superminds. Even when most part of the technology is already developed, we have holes in design and execution that put people away from policy development. We aim at changing this scenario by designing a new system with meta-structures based on digital tech, federated architectures, and AI. Current AI approaches may provide a certain depth in data analysis, but they lack the wideness, which may lead to decisions with an uncontrolled impact. Novel computational frameworks are required to manage complexity and public–private interactions.

Multi-scale governance implies thinking in organizations and places as part of complex systems where they interact with each other giving rise to collective actions. Collectiveness is not contradictory with organization, however, new multi-scale organization systems are required to manage the complexity. Leadership and networks are fundamental to build a more responsive and robust socioeconomic tissue. Leadership is required to drive change, but we need to consider two fundamental aspects in leadership: ideas and dynamics of the networks. We must open the chance for computational governance to complement existing mechanisms and allow disruption at scale.

This type of framework is not only good for existing structures and organizations, but it is also necessary for innovation and drive constructive efforts in the private sector, especially in ecosystems of start-ups. A new type of socioeconomic tissue is based on the collaboration that aligns collective efforts in specific challenges and missions (Mazzucato et al., 2020) with roper scientific and quantitative frameworks. From biological systems, we can learn that a certain level of specialization combined with interactions and sensing mechanisms is the foundation for emergent properties such as sustainability.

The human side of governance and policymaking cannot be overlooked. Collaborative efforts have to be integrated into governance platforms with capacities and capabilities to drive new technological revolutions. Technology should be the basis for activation, data sharing, collaboration, exchanges and also mediate in partnerships to catalyze responsible action.

Collaboration and coordination should be the ground for governance, always driven by AI and Data systems. These platforms need to introduce more disruptive elements based on organizational and technological innovation. Necessarily, this process must be undertaken laterally and vertically considering the local problems of people but incrementing the awareness of the systemic problems we face, including ethical frameworks. Sustainability goals must be achieved in an inclusive way (Omodei et al., 2022), so acceleration and inclusiveness must be coupled into the actions for new policies and governance platforms. This is not only an ethical principle, but a design, implementation, and deployment principle for an interconnected world that is resilient and sustainable. Ethical principles that promote protection, action, and future projection should be the basis of evidence-based systems (Pastor-Escuredo and Vinuesa, 2020) and also the right use of AI (Vinuesa et al., 2020a; Koshiyama et al., 2022).

Designing a societal system based on multi-scale governance will be a process that requires experimentation and space for failure also demanding robustness of the system to mitigate negative outcomes and impacts (Pastor-Escuredo et al., 2020). Data-intensive applications are risky in terms of privacy and competitive interests. Algorithmic governance (Treleaven and Batrinca, 2017; Engin and Treleaven, 2019; Rahwan et al., 2019) can help by quantifying the level of utility and risk of data to be used for decision-making within corporations and across corporations and sectors.

## Data availability statement

The original contributions presented in the study are included in the article/supplementary material, further inquiries can be directed to the corresponding author.

## Author contributions

DP-E led the collaboration and wrote the manuscript. AG, JK, AI, PT, and DP-E conceived, designed the research, and made the research. PT wrote the manuscript. All authors contributed to the article and approved the submitted version.

## Conflict of interest

Authors AG and JK were employed by CGIAR. AI was emloyed by United Nations High Commissioner for Refugees. The remaining authors declare that the research was conducted in the absence of any commercial or financial relationships that could be construed as a potential conflict of interest. The handling editor declared a past co-authorship with the author(s) DP-E.

## Publisher's note

All claims expressed in this article are solely those of the authors and do not necessarily represent those of their affiliated organizations, or those of the publisher, the editors and the reviewers. Any product that may be evaluated in this article, or claim that may be made by its manufacturer, is not guaranteed or endorsed by the publisher.
